# Reference biomechanical parameters and natural asymmetry among runners across the age spectrum without a history of running-related injuries

**DOI:** 10.3389/fspor.2025.1560756

**Published:** 2025-05-01

**Authors:** Heather K. Vincent, Reed Popp, Orlando Cicilioni, Kevin R. Vincent, Lydia Pezzullo, Matthew Martenson, Ryan M. Nixon

**Affiliations:** ^1^ Exercise and Functional Fitness Laboratory, Department of Physical Medicine and Rehabilitation, University of Florida, Gainesville, FL, United States; ^2^ Department of Applied Physiology and Kinesiology, University of Florida, Gainesville, FL, United States

**Keywords:** running, gait, biomechanics, interlimb asymmetry, ground reaction force (GRF)

## Abstract

**Introduction:**

Running biomechanics and expected mechanical asymmetries with no history of running-related injuries, and (2) determine whether age influenced gait parameter asymmetry.

**Methods:**

This cross-sectional study included 250 runners were used to test age effects on biomechanical variables and asymmetry outcomes. Effect sizes of age brackets were determined for runner characteristics and biomechanical variables.

**Results:**

Runners aged 55 years had the slowest velocity and 1.7%–4% slower occurred in ankle flexion moments, ankle and knee frontal excursions, and peak VALR (range, 12.1%–33.8% different between right and left limbs).

**Discussion:**

Given that we did not find consistent effects. These data can help inform reference ranges of normative biomechanical metrics and guide clinicians in gait retraining and performance targets across the age spectrum.

## 1 Introduction

Functional asymmetries and limb imbalance have become increasingly important in sports assessment due to potential associations with injury risk and performance loss (1). Tracking changes in asymmetry over time can be used to monitor rehabilitation progress and readiness to return to sports after injury. Asymmetry is the difference or disparity in function or performance of one limb relative to the other (1). For some sports motions, an interlimb asymmetry threshold of 10%–15% has been suggested to be “abnormal” (2), but this has not been validated across all sports. Growing evidence shows that asymmetries also exist in the biomechanical parameters of running (1, 3–9). Management of these imbalances, rather than complete mitigation of asymmetry, is important to reduce exposure to disproportionate loading and offset injury risk. For runners, there is not yet a consensus on what “normal” or expected levels of natural asymmetry are in running across the range of biomechanical parameters typically collected during gait analyses and whether these levels are different across the age spectrum. From this point forward, we have defined natural asymmetry as the interlimb differences in running parameters among healthy runners. This information is critical to identifying clinically meaningful biomechanical interlimb differences, improving gait analysis interpretation, and setting expectations for performance for runners across the lifespan.

The magnitude of interlimb biomechanical asymmetry may depend on the individual runner and the mechanical measure (3, 10). In adult runners, sagittal plane kinematic variables have low asymmetry (1%–9.7%) (3, 7, 10), whereas frontal motion asymmetries range widely from 12% to 39%. (3, 6) Kinetic, work-related, or load rate parameters have been characterized by a wide range of asymmetries from low to high (5%–35%) (3, 9). Translation of this evidence into general clinical settings is difficult, because of several methodological issues. First, injury histories may not be presented; if injuries are present during testing, these are at varying stages of acuity (8, 9). Second, other studies focused on collegiate athletes only (3, 7) or runners of younger age only (1). Third, some studies include other non-running-specific athletes (such as football, soccer, and basketball) with cross country and track runners as part of a combined asymmetry analysis (3). Thus, the asymmetry levels described in these earlier studies may not actually represent what occurs in the broader running-specific population. Furthermore, it is unclear whether these published asymmetries are expected to be the same across runners of varying ages.

The general running population has undergone an age-related expansion over the last 15 years. In 2021, estimates indicate that 21 million people aged 16–50 years old participated in running and jogging in the USA (11). The segments of runners aged 55–65 years and >65 years increased from 10.8% to 16% and from 2.6% to 9% during 2015–2022 (12). While we and others have studied basic differences between running motion in young and older runners (13–17), the nature and magnitude of biomechanical asymmetries have not been clearly established across the age continuum in runners without confounding injury histories. Therefore, the purposes of this study were to (1) produce reference running gait biomechanical data in healthy non-injured runners aged 15–75 years with no history of running injuries and (2) determine whether age influenced gait parameter asymmetry. We hypothesized that compared with older runners, younger runners would produce higher values for some spatiotemporal parameters, with larger lower body joint excursions and vertical stiffness, and higher values of kinetic parameters. We also hypothesized that younger runners, who may still be developing their neuromotor skills, would demonstrate higher natural interlimb asymmetry among spatiotemporal, kinematic, and kinetic variables compared with older runners.

## 2 Materials and methods

### 2.1 Study design

**Figure 1:**
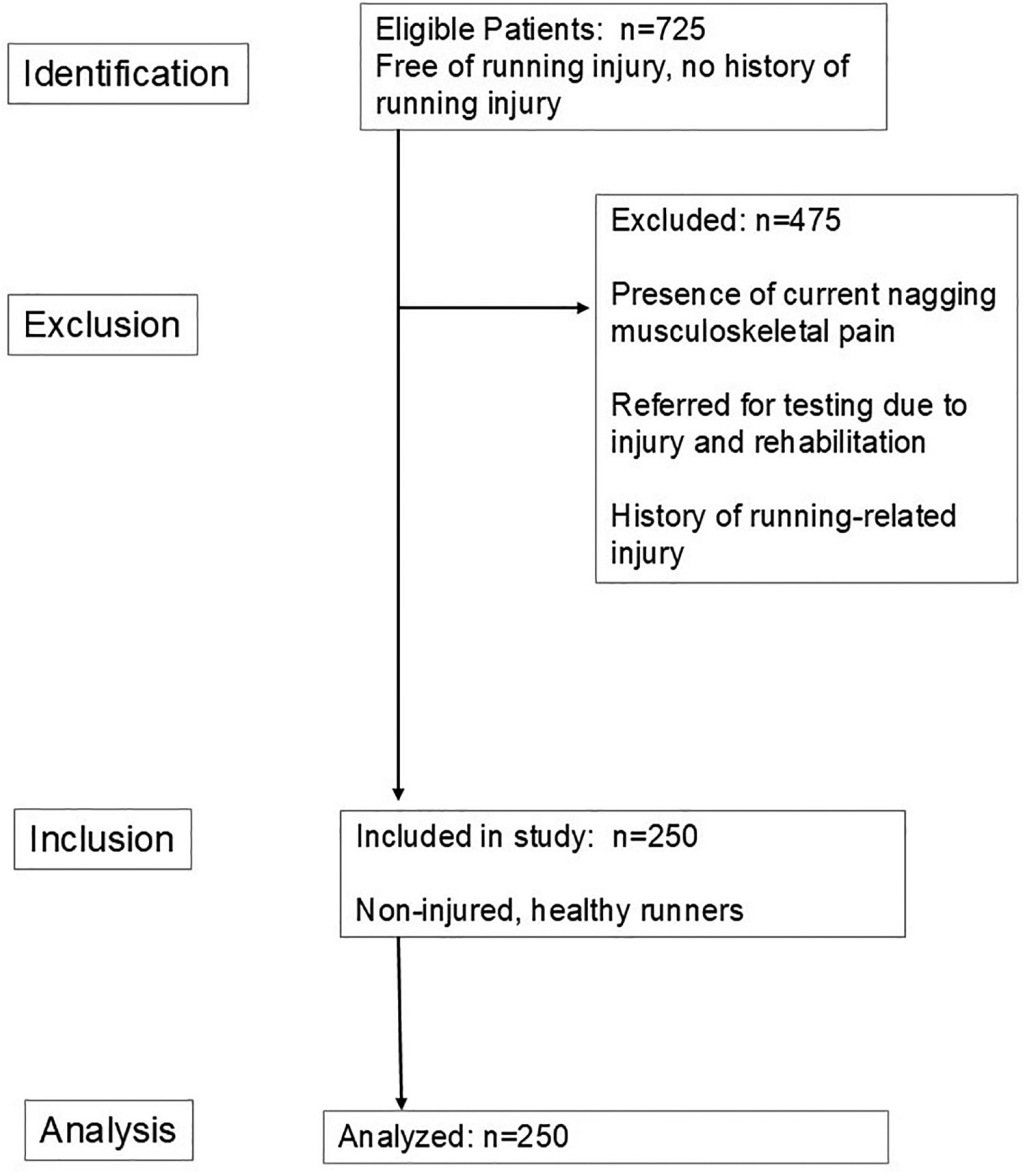
STROBE study flow diagram for observational studies.

This is a cross-sectional study of the habitual gait performance of endurance runners without running-related injuries. This study and its procedures followed the guidelines for the Declaration of Helsinki's Protection of Human Subjects. The study was approved by the University of Florida Institutional Review Board (IRB #202500475). The manuscript follows the recommended format for the observational study described by the statement in strengthening the reporting of observational studies in epidemiology (STROBE; which can be found in Supplementary Material) (18). The study flow diagram is shown in Figure 1.

### 2.2 Setting

The Exercise Medicine and Functional Fitness laboratory is located in a quaternary health care facility. Data from all runners who obtained running analyses for performance and injury prevention services were pulled for analysis from this setting from January 2014 to 20 March 2025 (*N* = 725).

### 2.3 Participants

Of all the runners who sought clinical gait services, a total of 250 had no self-reported history of running-related injuries. All runners provided written informed consent to place their data into our departmental research databank (IRB #202101632). Injury-free runners participating in high school cross country programs (aged 15–18 years) through young adult and masters' runners were eligible (aged 19–75 years). The inclusion criteria were as follows: male and female runners aged 15–75 years, who never incurred a running-related injury or injury with running sports. This age range was selected to represent the ages and proportions of running demographics nationwide (19). The exclusion criteria were as follows: (1) previous history or current presence of any running-related musculoskeletal injury, current or recent musculoskeletal pain due to running, irrespective of severity, (2) no major traumatic musculoskeletal injury or repeated chronic injuries (i.e., anterior cruciate ligament rupture, or repeated shin splints, chronic iliotibial band syndrome, patellofemoral pain) from running-related sports, and (3) other preexisting conditions that interfered with normal gait (i.e., scoliosis, anterior cruciate ligament repair, neurological conditions, or previous history or orthopedic trauma with resultant persistent motion aberrations). Runners were categorized into four groups by age: ≤18 years (high school), 19–35 years (collegiate, young adult), 36–55 years (master runners, younger bracket), and ≥55 years (master runners, older bracket). All had previous experience of >60 min of using treadmills as part of training, which is considered ample for treadmill accommodation (20).

#### 2.3.1 Characteristics

Characteristics were collected from a comprehensive intake form based on our published recommendations for runner assessment (21). Supplementary Table S1 provides the content collected on this intake form. Sections included characteristics and medical history, any pain symptoms, training history (volume, type, cross-training activities, strengthening exercise), shoe wear and orthotics if applicable (weight, heel-to-toe drop, heel height), and whether or not they were currently training for competition (yes, no). Each runner was permitted to use their habitual preferred shoes for the testing to minimize any acute effects of changing footwear on the kinematic and kinetic data.

### 2.4 Data sources and measurements

Data were acquired from a comprehensive health history intake and biomechanical running gait analyses.

#### 2.4.1 Initial procedures and instrumentation for running analysis

A standard procedure was performed in which motion capture during running at self-selected speed was captured using a high-speed, seven-camera 3D optical motion analysis system (Motion Analysis Corporation, Santa Rosa, CA, USA) that sampled at 200 Hz that was synchronized with force plate data collected from an instrumented treadmill (AMTI, Watertown, MA, USA) at 1,200 Hz (22, 23). Previously published methods were used to apply 33 retroreflective markers on anatomical landmarks (24). Markers were placed acromion processes, triceps, lateral elbows, radial forearms, dorsal wrists, posterior superior iliac spine, anterior superior iliac spine, anterior bilaterally on the thigh, medial and lateral condyles of the femur, tibial tuberosity, medial and lateral malleoli, calcaneus, lateral to the head of the fifth metatarsal, and medial to the base of the hallux. One offset marker was placed on the right scapular inferior angle.

Prior to data collection, a static calibration trial was performed to generate the computer model of each runner in the software in a neutral anatomical position (Cortex, Motion Analysis Corporation, Santa Rosa, CA, USA). The optical motion cameras captured the static positions of the retroreflective markers, and the software identified each marker in the baseline skeletal model based on the known marker set. The software then determined the body segment lengths and joint centers based on the anatomical marker placement. If any markers were misaligned or missed, this was corrected, and a new static trial was collected. The medial knee and ankle markers were then removed for all running conditions to avoid these being knocked off during testing.

#### 2.4.2 Data collection during running and post-processing

Runners ran at a self-selected velocity that was defined as a typical pace “used for long run distance training.” After an 8 min acclimation running period, slow-motion videos were captured for reference in the sagittal and frontal planes. Between 9 and 10 min, a 10 s sample of data was captured, with an average of 12–14 strides.

Joint angles at initial foot contact for the ankle, knee, hip, and pelvis were determined from the software tracking of the retroreflective markers in space in each frame for each trial. Using the foot markers, the ankle angle at the foot strike was calculated as the foot segment and the ground at initial contact relative to the natural angle during standing (25). The reference point for joint angles was established at 90° as vertical for the knee and hip and 0° as horizontal for the ankle angle. The pelvis was developed from the anterior and posterior superior iliac spine markers, and the anterior inclination was expressed relative to the horizontal as 0°of anterior tilt.

Several standard spatiotemporal spatial and kinematic variables were determined to produce the reference values of these measures by age bracket and to show the performance of this sample compared with other published evidence. Bone models were developed for each runner with the individual COM location using commercially available software (Visual3D, C-Motion, Inc.; Germantown, MD) (22, 26). Marker data were filtered at 9 Hz with a fourth-order, low-pass Butterworth filter. Bone models were created for every runner with an individual center of mass (COM) location in the methods of de Leva et al. (27). Gait cycle time is presented in percent (0% = initial foot contact, 100% = same foot contact post-swing phase). Cadence (steps/min) and the vertical displacement of the COM (the difference between the minimal and maximal vertical height of the COM during a gait cycle) were calculated. The distance between two successive placements of the same foot was defined as stride length. The medial–lateral distance between the proximal end position of the foot at the foot strike to the proximal end position of the foot at the next contralateral foot strike was calculated as stride width. Stance time was determined as the time that each foot was in contact with the treadmill. Variability of stride and step length, stride width, and stance time were determined as the standard deviation (SD) of the gait cycles collected. The medial–lateral range of motion (ROM) excursion of the COM was calculated as the shift in the medial–lateral positions of the estimated COM during an average gait cycle.

Foot strike type was determined by the angle between the foot segment and the horizontal ground at foot contact and was visually confirmed by the investigators with high-speed videos. Runners were categorized into rearfoot and non-rearfoot strikers. Joint ROM of the ankle, knee, and hip represented the angular excursion of the joint in the sagittal plane during an average gait cycle (flexion/extension motion for the ankle, knee, and hip). The pelvis was developed from the anterior and posterior superior iliac spine markers, and the anterior inclination was expressed relative to the horizontal as 0° of anterior tilt. Peak hip adduction angle (pelvic drop) was calculated from the angle created by the thigh segment and the pelvis.

Force data were collected from the instrumented treadmill at a frequency of 1,200 Hz. A threshold of 20 N ground reaction force (GRF) was used to set the initial foot contact and toe-off. GRF data were low-pass filtered at a frequency cutoff of 40 Hz using a fourth-order Butterworth filter. Processed, filtered treadmill data were integrated by Cortex software with the motion data, and three-dimensional kinetics were determined via full inverse dynamics calculations in Visual3D. The vertical component of the peak ground reaction force (GRF), the vertical average loading rate (VALR), and vertical impulses were normalized to body weight. The vertical component was chosen here as this is the most widely studied aspect of loading in the literature. VALR was calculated using previous methods from the slope of the Δ*F*/Δ*t* of the most linear portion of the force curve, where Δ*F* is the change of vertical force and Δ*t* is the change of time (between 20% and 80% of the first rise to the peak of the vertical GRF (28) or vertical GRF at 13% of stance in case the initial peak was absent. The area under the vertical GRF-time curve was the vertical impulses, which was calculated as the integral of vertical GRF over stance time (29). Vertical stiffness was estimated using the following: *K*_vert_ = *F*_max_/Δ*y*, where *F*_max_ is the peak vertical force and Δ*y* is the maximum displacement of the COM (30). The *K*_vert_ can estimate the neuromodulation of lower body activity (15) as it describes the interaction of the load placed on the leg and central nervous response and limb's responses to attenuate that load. The peak joint moments in the sagittal plane (flexion/extension) were determined for the ankle, knee, and hip. Joint moments were normalized to body mass multiplied by leg length. The preprocessed filtered treadmill data were combined with the motion data. Three-dimensional kinetics were determined via full inverse dynamics calculations implemented in Visual3D.

There are several methods for quantifying interlimb asymmetry (7, 31). Here, we express asymmetry as the absolute difference between the left and right limbs as raw data and as a percent difference between limbs with the right side as the comparison (3). This method was chosen to minimize the risk of error in determining sidedness, and it did not affect the calculation of absolute interlimb differences in biomechanical variables.

### 2.5 Statistical considerations

Statistical analyses were performed using SPSS version 29.0 (IBM, Armonk, NY, USA). Normality of the data (skewness, kurtosis), homogeneity of variance, and sphericity were assessed using Shapiro–Wilk's test, Levene's test, and Mauchly's test of sphericity, and descriptive statistics were calculated on all study variables and demographics. The assumptions of normality and analysis of variance (ANOVA) were performed on demographic, anthropometric, and training history continuous variables to determine if differences existed between by age bracket. Chi-square tests (*χ*^2^) were used to determine if there were differences in the categorical variables among the four age groups. Univariate analyses of variance with covariates (ANCOVA) were used to test group differences for biomechanical variables, where the between-group factor was age (≤18, 19–35, 36–55, and ≥55 years). Based on published evidence that running velocity and sex can affect the biomechanics of running (32), these variables were entered as covariates. The eta squared (*η*^2^) was provided to show the effect sizes for continuous variables; values of 0.01, 0.06, and 0.14 represented negligible to small, medium, and large effects, respectively (33). Phi values (*ɸ*) were determined as effect sizes for categorical variables. Statistical significance was established in advance at *p* < 0.05.

## 3 Results

### 3.1 Runner characteristics

Table 1 provides the characteristics of all four age brackets. Overall, the groups were well-matched for demographics and characteristics, with the expected difference in mean age and associated years of running experience. Weekly distance and regular participation in strength training were highest in the 36–55-year age bracket. Differences existed among groups with respect to participation in other types of cross-training. Runners aged 36–55 years participated most often in strength training, yoga/Pilates, CrossFit, and cycling, whereas runners aged >55 years used the elliptical trainer most often. Swimming was more often performed by runners aged <18 and 36–55 years (all *p* < 0.05). Runners aged <18 years had a higher prevalence of individual's sole activity was running (*p* < 0.001).

**Table 1:** Characteristics of runners.

Age brackets (year)	≤18	19–35	36–55	≥55	*p*	*η*^2^ or *ɸ*
	*n* = 29	*n* = 119	*n* = 74	*n* = 28		
Age (year)	15.8 ± 2.3	25.6 ± 4.9	44.2 ± 5.5	61.1 ± 5.1	<0.001	0.888
Sex (female #/%)	17 (58.6)	52 (43.7)	32 (43.2)	12 (42.9)	0.495	0.098
Height (cm)	168.2 ± 9.4	172.2 ± 10.4	172.9 ± 10.2	171.9 ± 10.2	0.194	0.019
Weight (kg)	64.6 ± 11.9	67.3 ± 12.6	69.2 ± 14.6	71.8 ± 17.5	0.198	0.019
BMI (kg/m^2^)	22.7 ± 2.9	22.6 ± 3.1	23.0 ± 3.7	23.4 ± 4.0	0.725	0.005
Competing (#/% yes)	20 (62.5)	86 (54.2)	63 (72.9)	17 (52.2)	0.358	0.326
Running experience (year)	9.5 ± 11.5	7.6 ± 6.9	10.1 ± 10.8	17.0 ± 15.8^b^	0.004	0.076
Runs per week (#)	3.8 ± 1.7	4.5 ± 1.9	4.4 ± 1.3	4.9 ± 3.5	0.284	0.016
Weekly distance (km)	32.0 ± 14.0	39.5 ± 18.9	43.1 ± 23.8^a^	35.2 ± 24.5	0.033	0.028
Current participation in other activities (%)						
Strength training	34.5	37.8	54.1^a^	39.3	<0.001	0.386
Yoga/Pilates	24.1	21.8	29.7	10.7	0.002	0.293
Cycling	34.5	28.6	48.6	32.1	0.001	0.294
Swimming	24.1	20.2	24.3	21.4	0.017	0.248
Elliptical	0.0	1.7	1.4	7.1	<0.001	0.402
CrossFit	6.9	0.0	4.1	3.6	<0.001	0.398
Running only	13.8	5.0	6.8	7.1	<0.001	0.380
Shoe wear characteristics						
Weight (oz)	9.6 ± 2.1	9.3 ± 3.4	8.9 ± 1.5	9.6 ± 1.5	0.470	0.011
Heel/toe drop (m)	7.5 ± 3.1	7.2 ± 3.3	8.3 ± 3.1	6.7 ± 3.9	0.098	0.027
Heel height (mm)	28.7 ± 9.4	29.0 ± 6.9	30.6 ± 5.8	30.9 ± 4.9	0.260	0.018
Rearfoot striker (#, %)	25 (86.2)	82 (69.5)	54 (73.0)	17 (63.0)	0.205	0.133

Means and standard deviation (SD) or % of the group are shown.

The mean age was different among all groups at *p* < 0.05.

^a^ Different than runners aged ≤18 years.

^b^ Different than runners aged ≤18, 19–35, and 36–55 years.

### 3.2 Select spatiotemporal parameters and variability

Table 2 provides the running velocities, temporal–spatial parameters, interlimb asymmetry, and variability about the means. Runners aged ≥55 years self-selected a running velocity and cadence lower than runners aged 19–35 and 36–55 years (both *p* < 0.05). The oldest age bracket also demonstrated the least asymmetry between limbs for step length (*p* = 0.02). COG vertical displacement, step width, and step times were not different by age bracket. The effect sizes for age bracket on these spatiotemporal variables as raw data, R–L asymmetry, and % differences were all found to be between small and moderate (*η*^2^ range, 0.000–0.040).

**Table 2:** Habitual velocity and selected temporal spatial parameters.

Age bracket (year)	≤18	19–35	36–55	≥55	*p*	*η* ^2^
Velocity (km/h)	9.8 ± 1.2 (9.4–10.2)	10.4 ± 1.5 (10.1–10.6)	10.2 ± 1.7 (9.8–10.6)	9.4 ± 1.6 (8.8–10.0)^a^	0.011*	0.040
Velocity (m/s)	2.7 ± 0.3 (2.6–2.8)	2.9 ± 0.4 (2.8–2.9)	2.8 ± 0.5 (2.7–2.9)	2.6 ± 0.4 (2.4–2.8)	0.011*	0.040
Cadence (step/min)	167 ± 9 (163–171)	171 ± 11 (168–172)	170 ± 9.2 (168–172)	164 ± 11 (159–167)^b^	0.011*	0.025
COG displacement (cm)						
Medial–lateral	2.4 ± 6.7 (2.2–2.7)	2.6 ± 7.5 (2.4–2.7)	2.6 ± 6.4 (2.5–2.8)	2.7 ± 7.3 (2.4–3.0)	0.493	0.020
Vertical	9.1 ± 1.3 (8.5–9.5)	9.1 ± 1.4 (8.8–9.3)	8.8 ± 1.3 (8.5–9.1)	9.1 ± 1.4 (8.6–9.7)	0.595	0.015
Step width (cm)						
Average	8.2 ± 2.4 (7.2–9.1)	8.8 ± 2.7 (8.3–9.3)	8.6 ± 2.4 (8.0–9.1)	9.0 ± 2.7 (8.0–10.0)	0.564	0.010
Variability	0.012 ± 0.003 (0.011–0.014)	0.012 ± 0.004 (0.011–0.013)	0.011 ± 0.004 (0.010–0.011)	0.012 ± 0.004 (0.011–0.014)	0.224	0.019
Step length (cm)						
Average	93.2 ± 12.5 (88.5–98.0)	97.5 ± 16.1 (94.5–100.4)	95.1 ± 13.6 (91.9–98.3)	90.9 ± 14.2 (85.3–96.3)	0.135	0.008
R–L asymmetry	2.2 ± 2.1 (1.3–2.8)	2.2 ± 1.8 (1.9–2.6)	1.5 ± 1.4 (1.3–1.9)	1.3 ± 1.1 (0.9–1.9)^c^	0.020*	0.036
Difference (%)	2.3 ± 2.5	2.3 ± 1.8	1.7 ± 1.5	1.6 ± 1.2^a^	0.026*	0.037
Step time						
Average (s)	0.72 ± 0.04 (0.71–0.74)	0.70 ± 0.05 (0.69–0.72)	0.71 ± 0.04 (0.70–0.72]	0.73 ± 0.04 (0.71–0.75)	0.321	0.015
R–L asymmetry (ms)	0.76 ± 0.63 (0.52–0.100)	0.75 ± 0.61 (0.65–0.86)	0.75 ± 0.72 (0.58–0.93)	0.80 ± 0.86 (0.45–1.14)	0.544	0.010
Difference (%)	2.1 ± 1.8	2.2 ± 1.8	2.1 ± 1.9	2.2 ± 2.3	0.488	0.000
Stance time (s)						
Average	0.27 ± 0.03 [0.26–0.28)	0.26 ± 0.03 (0.25–0.26)	0.27 ± 0.04 (0.26–0.27)	0.27 ± 0.03 (0.26–0.28)	0.065	0.040
R–L asymmetry	0.004 ± 0.003 (0.003–0.005)	0.005 ± 0.004 (0.004–0.006)	0.005 ± 0.004 (0.004–0.006)	0.006 ± 0.004 (0.005–0.008)	0.176	0.035
Difference (%)	3.7 ± 1.2	4.0 ± 1.1	3.5 ± 1.28^a^	3.7 ± 1.1	0.037*	0.020

Means ± SD (95% CI) are shown and are covaried for running velocity and sex. *Statistically significant.

^a^ Different than runners aged 19–35 years.

^b^ Different than runners aged 19–35 and 36–55 years.

^c^ Different than runners aged ≤18 and 19–35 years.

### 3.3 Kinetic features and vertical stiffness

Peak GRF, VALR, GRF impulses, and *K*_vert_ are provided for each age bracket in Table 3. The peak GRF values were lowest in runners aged ≥55 years and highest in runners aged 19–35 years (*p* = 0.027). The peak VALR values were 8%–18% lower in runners aged ≥55 years than those of the remaining groups, and the VALR interlimb asymmetry was lowest in the oldest two age brackets and highest in runners aged 19–35 years (*p* < 0.001). The *K*_vert_ values were 12.1% lower in runners aged >18 years than those of runners aged 36–55 years (*p* = 0.015). The effect sizes for the age bracket on most of these kinetic features and vertical stiffness as raw data, R–L asymmetry, and % differences were found to be between small and moderate (*η*^2^ range, 0.003–0.057). However, there was a moderate effect of age on peak VALR R–L symmetry (*η*^2^ = 0.083).

**Table 3:** Ground reaction forces (GRF), vertical average loading rate (VALR), leg vertical stiffness values (*K*_vert_), and associated interlimb asymmetry.

Age bracket (year)	≤18	19–35	36–55	≥55	*p*	*η* ^2^
Peak GRF (BW/s)						
Average	2.38 ± 0.32 (2.25–2.50)	2.56 ± 0.34 (2.50–2.62)	2.44 ± 0.44 (2.33–2.54)	2.3 ± 0.29 (2.18–2.41)^c^	0.026*	0.057
R–L asymmetry	0.05 ± 0.04 (0.04–0.07)	0.07 ± 0.05 (0.06–0.08)	0.06 ± 0.04 (0.05–0.07)	0.07 ± 0.03 (0.05–0.08)	0.286	0.016
Difference (%)	2.3 ± 1.7	2.7 ± 2.0	2.4 ± 1.8	3.1 ± 1.7	0.423	0.011
Peak VALR (BW/s)						
Average	63.1 ± 17.7 (56.4–69.9)	70.5 ± 25.6 (65.8–75.2)	63.5 ± 23.0 (59.5–71.2)	57.8 ± 21.5 (48.9–66.7)	0.193	0.019
R–L asymmetry	12.5 ± 10.3 (8.6–17.4)	13.9 ± 11.2 (11.9–15.9)^b^	7.6 ± 6.5 (6.1–9.1)	9.3 ± 7.1 (7.1–12.7)	<0.001*	0.083
Difference (%)	20.0 ± 18.8	20.2 ± 16.8^b^	12.1 ± 9.6	20.3 ± 16.9	0.003*	0.057
GRF impulse (BW/s)						
Average	0.37 ± 0.03 (0.36–0.38)	0.40 ± 0.14 (0.37–0.42)	0.38 ± 0.05 (0.37–0.39)	0.37 ± 0.03 (0.36–0.38)	0.307	0.015
R–L asymmetry	0.01 ± 0.01 (0.01–0.10)	0.01 ± 0.01 (0.01–0.10)	0.01 ± 0.01 (0.01–0.10)	0.01 ± 0.01 (0.01–0.10)	0.854	0.003
Difference (%)	2.7 ± 2.2	2.2 ± 2.1	2.3 ± 1.6	2.7 ± 2.0	0.633	0.007
*K*_vert_ (*N*/cm)						
Average	167 ± 28 (156–177)	186 ± 35 (179–192)	190 ± 40 (180–200)^a^	172 ± 32 (159–185)	0.011*	0.046
R–L asymmetry	3.9 ± 2.9 (2.7–5.0)	5.2 ± 4.2 (4.4–5.9)	4.7 ± 2.9 (3.9–5.7)	5.3 ± 3.2 (3.9–6.6)	0.524	0.009
Difference (%)	2.3 ± 1.7	2.7 ± 2.0	2.5 ± 1.8	3.0 ± 1.7	0.622	0.007

Means ± SD (95% CI) are shown and are covaried for running velocity and sex.

R–L, right to left; difference (%), difference of asymmetry expressed as percent of the average value. *Statistically significant.

^a^ Different than runners aged ≤18 years.

^b^ Different than runners aged 36–55 years.

^c^ Different than runners aged 19–35 years.

### 3.4 Joint excursions during a gait cycle

**Figure 2:**
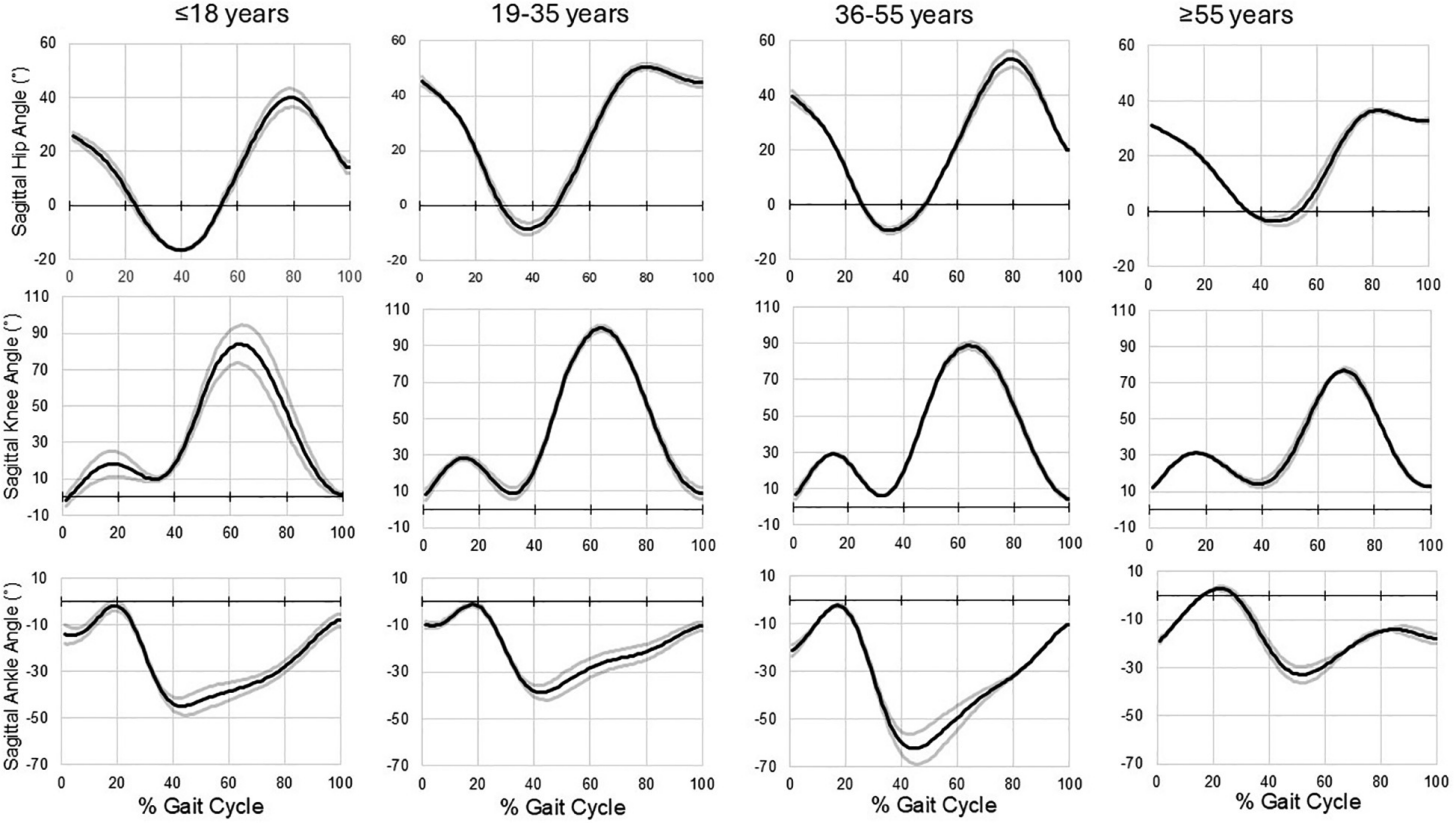
Sagittal plane waveform data during an average gait cycle are shown for the ankle, knee, and hip joints for all four age brackets. Values are expressed in degrees and are presented as mean and standard deviation envelopes.

Table 4 provides the overall joint excursions or ROM about the ankle, knee, hip, and pelvis in the sagittal and frontal planes. While overall ROM did not differ by age bracket for any peripheral joint, the contralateral pelvic drop was lowest in runners aged ≥55 years and highest in runners aged 36–55 years (*p* = 0.020). Interlimb asymmetry was highest in runners aged ≤18 years for knee flexion/extension (sagittal plane; *p* = 0.050), and knee adduction/abduction asymmetry tended to be higher in runners ≤18 and 36–55 years (*p* = 0.065). Overall, the percent differences were larger for frontal plane asymmetries compared with sagittal asymmetries. The effect size of age bracket on these joint excursion values was all considered to be small (*η*^2^ range, 0.003–0.029). Sagittal plane waveform data for the ankle, knee, and hip joints for all four age brackets are shown in Figure 2. Visually, the greatest ankle and hip excursions occurred in the 36–55-year group, whereas the least ankle and hip excursions occurred in the ≥55-year group.

**Table 4:** Average joint excursion during an average gait cycle and associated interlimb asymmetry. Values are expressed in degrees or percent difference.

Age bracket (year)	≤18	19–35	36–55	≥55	*p*	*η* ^2^
Sagittal						
Ankle	50.9 ± 6.4 (48.5–53.4)	49.3 ± 6.1 (48.2–50.4)	49.6 ± 7.3 (47.8–51.4)	48.4 ± 7.3 (45.5–51.4)	0.429	0.008
R–L asymmetry	2.9 ± 2.5 (1.9–3.8)	4.0 ± 3.3 (3.4–4.6)	4.1 ± 4.3 (3.1–5.1)	3.1 ± 2.6 (2.0–4.2)	0.142	0.023
Difference (%)	5.7 ± 4.8	8.2 ± 6.5	8.2 ± 7.4	6.7 ± 6.2	0.115	0.024
Knee	82.5 ± 10.4 (78.6–86.5)	83.4 ± 12.4 (81.2–85.7)	81.1 ± 11.4 (78.4–83.8)	80.1 ± 13.2 (74.8–85.5)	0.416	0.012
R–L asymmetry	4.7 ± 3.1 (3.5–5.9)^a^	3.7 ± 2.6 (3.2–4.1)	3.0 ± 2.6 (2.4–3.6)	3.7 ± 3.5 (2.3–5.1)	0.050*	0.029
Difference (%)	5.8 ± 4.2	4.6 ± 3.5	3.8 ± 3.3	4.6 ± 4.2	0.150	0.022
Hip	54.4 ± 6.3 (52.1–56.8)	54.6 ± 7.8 (53.2–55.9)	53.9 ± 6.2 (52.4–55.4)	51.1 ± 7.4 (48.1–54.1)	0.467	0.008
R–L asymmetry	3.2 ± 2.8 (2.1–4.2)	3.0 ± 2.0 (2.6–3.3)	3.2 ± 2.2 (2.6–3.7)	2.3 ± 2.4 (1.4–3.3)	0.437	0.010
Difference (%)	6.1 ± 6.1	5.6 ± 3.9	5.9 ± 4.3	4.7 ± 4.9	0.532	0.009
Pelvis	7.8 ± 2.0 (7.1–8.6)	8.1 ± 2.2 (7.7–8.6)	8.2 ± 1.8 (7.7–8.6)	7.7 ± 2.2 (6.8–8.6)	0.856	0.003
Frontal						
Ankle	15.3 ± 3.2 (14.1–16.5)	15.1 ± 4.9 (14.3–16.90)	14.8 ± 4.1 (13.9–5.8)	15.4 ± 4.0 (13.7–17.0)	0.913	0.003
R–L asymmetry	2.0 ± 1.2 (1.5–2.4)	3.0 ± 2.8 (2.5–3.5)	2.9 ± 2.9 (2.2–3.6)	3.1 ± 2.6 (2.1–4.2)	0.294	0.015
Difference (%)	13.8 ± 9.7	19.4 ± 17.2	20.5 ± 20.7	20.1 ± 14.1	0.397	0.012
Knee	10.0 ± 3.8 (8.6–11.5)	9.8 ± 3.1 (9.2–10.3)	10.6 ± 4.8 (9.4–11.7)	9.2 ± 2.8 (8.1–10.4)	0.360	0.012
R–L asymmetry	3.3 ± 3.6 (1.9–4.7)	2.5 ± 1.9 (2.2–2.9)	3.4 ± 3.0 (2.7–4.2)	2.5 ± 1.7 (1.8–3.2)	0.065	0.029
Difference (%)	41.0 ± 63.1	26.4 ± 19.3	33.5 ± 29.9	28.8 ± 23.6	0.079	0.028
Hip	20.9 ± 5.1 (18.9–22.9)	20.7 ± 4.9 (19.8–21.6)	22.1 ± 5.6 (20.8–23.5)	19.3 ± 5.2 (20.8–23.5)	0.111	0.024
R–L asymmetry	1.8 ± 1.5 (1.2–2.4)	1.3 ± 1.2 (1.1–1.6)	1.6 ± 1.4 (1.3–2.0)	1.2 ± 0.8 (0.9–1.5)	0.123	0.025
Difference (%)	10.5 ± 10.4	7.6 ± 8.9	8.2 ± 7.4	6.8 ± 4.6	0.249	0.017
Pelvis	11.8 ± 3.1 (10.7–13.0)	11.8 ± 3.1 (11.3–12.4)	12.9 ± 3.1 (12.2–13.6)	10.7–2.8 (9.6–11.9)^a^	0.020 *	0.039

Means ± SD (95% CI) are shown and are covaried for running velocity and sex.

R–L, right to left; difference (%), difference of asymmetry expressed as percent of the average value. *Statistically significant.

^a^ Different than runners aged 36–55 years.

### 3.5 Joint moments

Joint moments for the ankle, knee, and hip in the sagittal plane are shown in Table 5. Overall, the average joint moments were not found to be statistically different by age group. Similarly, the percent differences in R–L asymmetries were not different by age group for any joint. Among the R–L asymmetries, only ankle flexion moment R–L asymmetry was significantly higher in the ≤18-year group than in the remaining age groups (*p* < 0.05). For all age groups, the largest percent differences in asymmetry occurred with ankle flexion (values ranged from 28.1% to 38.1%). The effect size of age bracket on these joint moments, irrespective of how these were expressed (raw, R–L asymmetry, or percent difference), were all found to be small (*η*^2^ range, 0.002–0.032).

**Table 5:** Joint moments during an average gait cycle.

Age bracket (year)	≤18	19–35	36–55	≥55	*p*	*η* ^2^
Maximum joint moment (Nm/kg) extension						
Ankle	2.66 ± 0.68 (2.40–2.92)	2.51 ± 0.53 (2.41–2.61)	2.53 ± 0.54 (2.40–2.66)	2.52 ± 0.55 (2.29–2.74)	0.300	0.015
R–L asymmetry	0.16 ± 0.15 (0.11–0.22)	0.16 ± 0.14 (0.14–0.19)	0.14 ± 0.13 (0.11–0.17)	0.14 ± 0.11 (0.09–0.18)	0.530	0.009
Difference (%)	6.4 ± 5.8 (4.2–8.6)	6.5 ± 5.6 (5.5–7.5)	5.3 ± 4.7 (4.2–6.5)	5.5 ± 4.2 (3.8–7.2)	0.415	0.012
Knee	2.23 ± 0.66 (1.98–2.49)	2.17 ± 0.69 (2.05–2.30)	2.07 ± 0.77 (1.89–2.49)	2.19 ± 0.74 (1.89–2.50)	0.241	0.017
R–L asymmetry	0.31 ± 0.19 (0.24–0.39)	0.26 ± 0.23 (0.22–0.30)	0.25 ± 0.17 (0.21–0.29)	0.21 ± 0.17 (0.14–0.27)	0.190	0.020
difference (%)	15.7 ± 11.0 (11.5–19.9)	13.2 ± 12.0 (11.0–15.4)	14.4 ± 14.8 (10.9–17.8)	8.7 ± 6.8 (5.9–11.5)	0.072	0.029
Hip	2.42 ± 0.79 (2.12–2.72)	2.54 ± 0.74 (2.41–2.67)	2.49 ± 0.78 (2.30–2.68)	2.52 ± 0.42 (2.35–2.69)	0.702	0.006
R–L asymmetry	0.26 ± 0.17 (0.19–0.32)	0.24 ± 0.22 (0.20–0.28)	0.22 ± 0.24 (0.16–0.28)	0.23 ± 0.19 (0.15–0.31)	0.782	0.004
Difference (%)	12.5 ± 9.9 (8.7–16.2)	9.1 ± 7.0 (7.8–10.3)	8.7 ± 7.4 (6.9–10.4)	8.9 ± 6.9 (6.1–11.7)	0.134	0.023
Minimum joint moment (Nm/kg) flexion						
Ankle	–0.14 ± 0.10 (–0.18 to –0.10)	–0.11 ± 0.10 (–0.13 to –0.09)	–0.11 ± 0.11 (–0.13 to –0.08)	–0.13 ± 0.11 (–0.17 to –0.08)	0.135	0.023
R–L asymmetry	0.06 ± 0.06 (0.03–0.08)^a^	0.04 ± 0.05 (0.03–0.05)	0.04 ± 0.04 (0.03–0.04)	0.04 ± 0.04 (0.02–0.06)	0.048*	0.032
Difference (%)	38.1 ± 40.7 (21.9–54.2)	33.9 ± 40.4 (26.4–41.4)	28.1 ± 27.5 (21.4–34.8)	35.2 ± 47.1 (15.7–54.6)	0.586	0.008
Knee	–0.72 ± 0.21 (–0.79 to –0.63)	–0.77 ± 0.25 (–0.82 to –0.72)	–0.75 ± 0.22 (–0.81 to –0.70)	–0.75 ± 0.16 (–0.82 to –0.68)	0.938	0.002
R–L asymmetry	0.08 ± 0.11 (0.04–0.13)	0.10 ± 0.14 (0.08–0.13)	0.11 ± 0.16 (0.07–0.15)	0.11 ± 0.12 (0.06–0.16)	0.867	0.003
Difference (%)	10.5 ± 10.8 (6.4–14.5)	12.2 ± 11.7 (10.1–14.3)	12.9 ± 13.7 (9.6–16.2)	12.7 ± 10.0 (8.7–16.8)	0.844	0.003
Hip	−1.21 ± 0.78 (−1.51 to –0.91)	−1.62 ± 1.18 (−1.57 to −1.08)	−1.33 ± 1.03 (−1.57 to −1.08)	−1.42 ± 1.05 (−1.87 to –0.99]	0.189	0.020
R–L asymmetry	0.12 ± 0.18 (0.05–0.19)	0.15 ± 0.18 (0.12–0.18)	0.13 ± 0.17 (0.09–0.17)	0.15 ± 0.19 (0.07–0.23)	0.767	0.005
Difference (%)	8.3 ± 6.3 (5.9–10.7)	8.7 ± 7.2 (7.4–10.0)	9.0 ± 8.3 (7.1–11.0)	7.9 ± 5.5 (5.7–10.3)	0.909	0.002

Means ± SD (95% CI) are shown and are covaried for running velocity and sex. *Statistically significant.

^a^ Different than all other groups at *p* < 0.05.

### 3.6 Asymmetries ranked by biomechanical parameter

**Figure 3:**
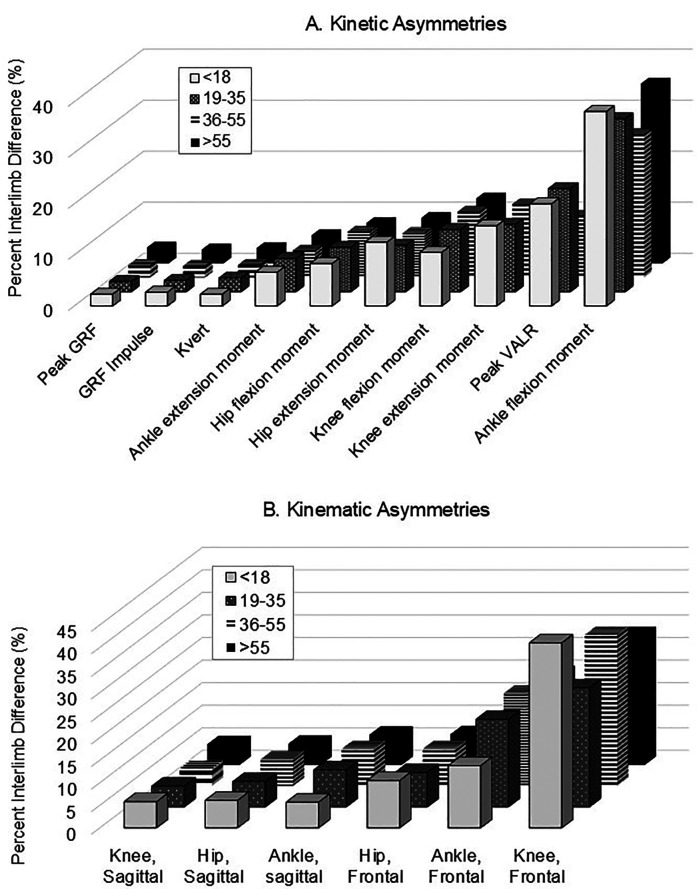
Mean interlimb percent asymmetries for biomechanical parameters by age, ranked from least to greatest. **(A)** The mean right-to-left percent asymmetry values for the kinetic and *K*_vert_ parameters. **(B)** The mean right-to-left percent asymmetry values for the kinematic variables.

Figures 3A,B show the average percent interlimb asymmetries for each age group ranked by parameter from least to greatest. Figure 3A provides the kinetic features and *K*_vert_, and the highest asymmetry occurred in the ankle flexion moment. Peak GRF values, GRF impulses, and *K*_vert_ values were found to have the lowest percent interlimb differences. Most joint moments were characterized by moderate interlimb differences, and the highest interlimb differences were consistently detected across age groups for peak VALR and ankle flexion moment. Figure 3B plots the percent interlimb asymmetries for kinematic variables. Sagittal joint motion interlimb differences were found to be lower than frontal joint motion. The parameter with the highest interlimb percent difference value was knee frontal excursion (knee adduction/abduction). The ≤18-year group had 7.5%–14.6% greater differences in knee frontal motion than the other three age groups (*p* = 0.050).

## 4 Discussion

The two main findings of this biomechanical analysis of injury-free healthy runners were as follows: the magnitude of interlimb asymmetry varied widely based on joint and parameter, and asymmetry was not consistently different by age group. We found that spatiotemporal asymmetries were generally low at <4% among all runners, irrespective of age. Depending on the joint and age group, lower extremity joint kinematics in the sagittal and frontal planes varied widely from 3.8% to 41%, with the highest asymmetry in knee abduction among runners aged ≤18 years. Some kinetic asymmetries such as ankle flexion moment and VALR varied widely from 12.1% to 38.1% independent of age. Overall, natural interlimb biomechanical asymmetry can be very high even without injury history or current pain, especially among kinetic parameters.

### 4.1 Age and running mechanics

In the present study, kinetic parameters, joint excursions, and spatiotemporal values were not consistently or progressively different with advancing age. Our biomechanical values overall are comparable to those reported in other studies in adults (32, 34, 35). At preferred velocities, older runners run at slower velocities (13) with lower peak vertical GRF than younger runners (13). Previous evidence shows that higher peak *K*_vert_ occurs in runners aged 60–70 years compared with runners aged 30–40 years while running at a standardized velocity (15). Our data do contrast with other studies that show progressively lower joint excursions for the ankle and hip and lower joint moments at the ankle, knee, and hip with advancing age (36). Our runners produced very similar joint excursions and joint moments as younger runners. There could be a few mechanisms related to this finding. First, the adoption of slightly slower running velocities and consistent maintenance of a high volume of running over years by older runners may help attenuate age-related declines in lower extremity joint function and skeletal muscle structure (37). In support of this point, our runners aged ≥55 years were running similar weekly distances with nearly twice the years of experience of runners aged ≤18 years. Hence, the ability of aging runners to maintain similar motion characteristics with high running volume supports biomechanical plasticity to better maintain running kinetics at the ankle, knee, and hip over time (38). Second, older runners may use different muscle activation patterns to dampen GRF and VALR, while controlling *K*_vert_ and preserving several temporal–spatial parameters, hip obliquity, and joint excursions similar to young runners. For example, co-activation of leg muscles (medial gastrocnemius) is higher in older runners during pre-activation through braking, likely to counteract aging-related changes in fascicle–tendon behaviors of the leg muscles (39). Third, maintenance of vigorous exercise enables the body to adapt to the consequences of age-related denervation and preserves muscle structure and function by stimulating muscle fibers through recruitment to different slow motor units (40).

### 4.2 Asymmetries in running motion across the age span

Comparative data from smaller studies of healthy runners with different experience levels and injury histories have also reported running parameter asymmetries (*N* range, 11–62 participants, young adults) (6, 9, 10, 15, 32, 41–43). Two larger studies (*N* = 210 and 836) that combined injured and non-injured runners for analyses found that interlimb spatiotemporal and kinetic variable asymmetries did not correspond to running injuries and elite runners had less asymmetry than novice runners (34, 35). When taken in context with these other studies, our main findings show that age itself was not related to the magnitude of natural interlimb asymmetry across most biomechanical measures. A recent systematic review that examined interlimb asymmetries in young adult middle and long-distance runners revealed consistently greater asymmetry among kinetic variables (2.6%–19.7%) compared with kinematic parameters (44). Preexisting morphological asymmetry in bone and segment lengths, neuromotor paths, motor units, or muscle mass can contribute to the natural asymmetries in running motion for runners, where greater interlimb anatomic differences correlate to kinematic asymmetry (45). Performance disadvantages can occur with interlimb differences in *K*_vert_, stance time, and ankle dorsiflexion angle that include higher energy cost and worse running times (44). However, there is a consideration that natural preexisting laterality (preference to use one side of the body for specific tasks), when coupled with anatomical variations, can produce asymmetries in running gait that are not necessarily linked to injury risk (46).

In contrast to our hypotheses, asymmetries were not consistently and linearly different by progressive age bracket. Younger runners (≤18 years) generated higher asymmetries with peak ankle flexion moment and frontal plane knee joint excursion compared with the other age brackets, but lower asymmetry with step length. Runners aged ≥55 years had lower asymmetry with step lengths than other age brackets. Similar to other reports (3, 10, 34), we found that symmetry in general appears to be more related to the type of parameter itself and less on age. Specifically, asymmetry in spatiotemporal parameters such as step times, step length, and stance times were low, whereas interlimb differences in joint excursions and kinetic parameters were low to very high. The ankle plantarflexion moments and knee frontal excursion were both highly asymmetric relative to other metrics. Prior studies have also found relatively low asymmetry in joint angles during stance in the sagittal plane (3, 6, 7, 10, 42) and higher knee joint motion asymmetry in the frontal plane (7). Others have shown that VALR and vertical GRF are characterized by relatively high asymmetries ranging from 14.1% to 20% (3, 32). Joint moments range in asymmetry from 5.5% to 22.0% (3), with *K*_vert_ interlimb differences at 9% (6). Studies that report individual runner biomechanical responses also show considerable variance within each measure, with the highest variation occurring in peak hip and knee moments (32). It is important to note that previously published data collection methods vary significantly compared with the present study, and this variation creates difficulty in contextualizing findings across studies (44). Specifically, overground runways, instrumented treadmills, runner experience, pressure measuring insoles, and motion capture were all used. Moreover, inconsistencies existed in testing protocol running velocities, cadences, and biomechanical outcomes. Despite these methodological differences, our data ranges are in similar ranges to published work.

### 4.3 Clinical implications

There are currently no published guidelines on what is considered “acceptable” or naturally occurring levels of asymmetries across runners of different ages. This has limited clinicians to extrapolating evidence from other sports activities and tasks to runners, which have used a 10%–15% interlimb functional asymmetry threshold as “abnormal” (2). Afonso et al. (46) suggested that asymmetries should be considered in a sports-specific context. For runners, we suggest here that irrespective of runner age, clinicians, and performance professionals may consider that higher natural asymmetry exists for some kinetics and frontal plane joint moments (4%–38%) and VALR (up to 20% or more) to and frontal plane motion (8%–41%), but lower asymmetries occur among spatiotemporal parameters (<5%), sagittal plane joint motion (less than 10%), and sagittal plane moments (up to 15%). After clinical gait analysis, these ranges may help clinicians determine kinetic chain targets for rehabilitation. Gait retraining, adoption of cues to improve form, and participation in therapeutic exercise can be focused on areas along the kinetic chain that are well outside the ranges we observed here. If runners can manage asymmetry values at these levels or lower, this may define whether “successful” rehabilitation outcomes were achieved. We acknowledge that it is not realistic or necessary to fully eliminate biomechanical asymmetries across all measures. However, a positive effect on comfort and load dissipation may be quickly achieved with even some correction of asymmetries (47). Additional evidence is needed for runners as to whether even small symmetry improvements correspond to injury onset or performance over the long term. These data can be used as healthy comparative reference values for future prospective studies of injury onset and fatigue effects in other runner types, such as trail runners, sprinters, and interval runners (runners who routinely train using alternating running and walking intervals), and ultra-endurance athletes.

### 4.4 Limitations, strengths, and future directions

This study has both limitations and strengths. This analysis was comprised of runners tested at one site at a quaternary care setting, but participants traveled from all over the state and out of state for services. The cohort profile was closely representative of the global runner population previously described (12), and as such, the findings are generalizable to the running greater running community. We do not have histories of other sports-related injuries that could have persistent effects on gait asymmetry or histories of other previous major surgeries that might have caused persistent impacts on joint kinematics. Moreover, it is not clear how different combinations or types of cross-training activities impact the mean values of gait parameters or asymmetry. All participants wore their own running shoes, which comprised different features and wear patterns that could contribute to the average values and degrees of asymmetry. The strengths of the study include a large sample size and the same testers and equipment used for all assessments. The present study improved the characterization of reference biomechanics through several methods. First, a large sample of runners with varying ages was enrolled with no confounding running-related injury history. Second, there was comprehensive reporting of spatiotemporal, kinematic, and kinetic outcomes at self-selected running velocities which may help approximate realistic running motion. Third, our choice to use joint excursions during a whole gait cycle, rather than discrete joint positions at initial foot contact, also provided critical insight into movement control along the lower extremity kinetic chain. Each age group was free of running injury history, which enables other researchers to have access to “clean” data specifically among endurance runners for future comparison.

As this area of research expands, it will be important to consider new powerful methods to analyze specific running gait features that could ultimately be predictive of “healthy” and “injury-prone” populations. For example, Xu et al. (48) used principal component analysis to identify which gait features contribute most to gait pattern recognition and provided a new method of metaheuristic optimization for realizing the optimal gait feature selection among high- and low-volume distance runners. This type of analysis may offer sports and clinical researchers deeper insight into their populations of study and thereby may help drive recommendations and injury prediction models.

### 4.5 Conclusion

These findings can be used as healthy comparative reference values for future studies and for helping guide clinicians on running rehabilitation goals and defining success for achieving a healthy gait. We propose that R–L kinetic or frontal plane joint moment asymmetries that exceed 4% up to 38%, loading rate asymmetries that exceed 20%, and frontal plane motion asymmetries that exceed 8%–41% may indicate the need for retraining methods to reduce these values. We also propose that asymmetries in spatiotemporal parameters, sagittal plane excursions that are >10%, and sagittal plane joint moments that exceed 15% may also indicate the need for retraining and running form modification. These values may be extended across the age spectrum.

## Data Availability

The raw data supporting the conclusions of this article will be made available by the authors, without undue reservation.
