# Supplementary material for: Reference biomechanical parameters and natural asymmetry among runners across the age spectrum without a history of running-related injuries
Source: Front Sports Act Living. 2025 May 1;7:1560756. doi: 10.3389/fspor.2025.1560756 (PMC12078198; doi:10.3389/fspor.2025.1560756)
Supplement: Supplementary file 1 [file Table1.docx]

Supplementary Table 1. Medical and training history factors to be reviewed with the injured runner. Please cite this paper for use.

Table modified from Vincent HK, Vincent KR. Evaluation of the injured runner. In: *Clinical Care of the Runner: Assessment, Biomechanical Principles, and Injury Management*. Elsevier; 2020:19-26.

**Question Item Additional Questions**

**Characteristics and Medical history**

Demographics Age, height, weight, body mass

index

Self-reported rankings (1-10, 1=poor and 10=excellent) Endurance, recovery, speed,

strength, flexibility, pain tolerance, motivation

Past and current medical conditions Menstrual history

Previous running injuries

Has health status changed

over last years?

Any previous running injuries? What were they?

When did they occur?

What was medical treatment plan?

Is any pain left from that injury?

Reason for engaging in running Fitness, weight management, social

reasons, stress management,

competition, other

**Any Pain Symptoms**

Where is the pain, how intense? Does pain worsen during running?

What kind of pain is it?

Does it get worse after running?

How long have you had the pain?

What makes the pain worse? List

What makes the pain better? List

Does the pain make you change your running gait Yes or No

or limp?

Have other pains developed since this one? If yes, what were these and when did

these appear?

How severe are these new pains?

**Training History**

How long have you been running? Months or years

Training status Recreational, competitive, elite,

other

Type of runner Endurance

Sprinter

Both endurance and sprinter

Interval runner (walk-run intervals)

Current training practices Weekly session number

Distance per week

Running session number per week

Average distance for a long run

Typical running surface and route description Beveled roads, asphalt, sand, beach, trail, treadmill, sidewalk, track

Same route and direction used all the time?

Currently doing speed work? If yes, how many times a week?

Currently training for a race? If yes, what distance and when is it?

Any recent changes to your routine? Select all that apply:

New running group

Increased mileage (more than 10% per week?)

New shoes or new inserts

Planning for a new race

Training in new terrain

Did you add speedwork or hills

Added speedwork to the routine

Changes in running volume, frequency, terrain or surface, If yes, what were they?

Have you changed shoes in the last six months? If yes, what specific change

What is your perceived foot strike pattern Rearfoot

Midfoot

Forefoot

Don’t know

How long have you had this strike pattern? List

Have you purposely tried to change your foot Yes or No

strike type over the last 6 months?

Do you run for exercise only? If no, what other endurance exercise

do you do regularly?

Other training in addition to running If yes, describe specific mode

(cycling, swimming, soccer,

basketball, CrossFit, elliptical, stairs, lacrosse, softball or baseball, golf, yoga/ Pilates, other)

Do you perform regular strength training? Yes or No

If yes, how often do you strength train? Weekly times and minutes

performed

What is the most common mode of strength training Machines

performed? Free weights

Body weight based

Are you a novice runner or have slow running time? Yes or No

**Shoe Wear and Orthotics**

Current shoe make and model Did you get advice on the shoe to choose?

What was reason for picking your shoe?

Shoe weight (oz)

Heel to toe drop (mm)

Heel height (mm)

Estimated mileage on current shoe Miles

Do you regularly use orthotics or inserts? What type are these (over the counter, full length, ¾ length, rigid

semi-rigid, custom, other)

How long have you used them?

What is the reason for use?

When did you make the change?

Do you wear poorly fitting, tight or worn shoes? Yes or No

Have you changed from a standard running shoe to If yes, when?

minimalist shoes or barefoot?
